# The evolution of research on depression during COVID-19: A visual analysis using Co-Occurrence and VOSviewer

**DOI:** 10.3389/fpubh.2022.1061486

**Published:** 2022-12-06

**Authors:** Qiannan Fu, Jiahao Ge, Yanhua Xu, Xiaoyu Liang, Yuyao Yu, Suqin Shen, Yanfang Ma, Jianzhen Zhang

**Affiliations:** ^1^College of Geography and Environmental Sciences, Zhejiang Normal University, Jinhua, China; ^2^College of Teacher Education, Zhejiang Normal University, Jinhua, China; ^3^School of Geography and Environment, Jiangxi Normal University, Nanchang, China

**Keywords:** COOC analysis, VOSviewer, depression during COVID-19, bibliometric analysis, visualization analysis

## Abstract

**Background:**

The COVID-19 pandemic has led to public health problems, including depression. There has been a significant increase in research on depression during the COVID-19 pandemic. However, little attention has been paid to the overall trend in this field based on bibliometric analyses.

**Methods:**

Co-Occurrence (COOC) and VOSviewer bibliometric methods were utilized to analyze depression in COVID-19 literature in the core collection of the Web of Science (WOS). The overall characteristics of depression during COVID-19 were summarized by analyzing the number of published studies, keywords, institutions, and countries.

**Results:**

A total of 9,694 English original research articles and reviews on depression during COVID-19 were included in this study. The United States, China, and the United Kingdom were the countries with the largest number of publications and had close cooperation with each other. Research institutions in each country were dominated by universities, with the University of Toronto being the most productive institution in the world. The most frequently published author was Ligang Zhang. Visualization analysis showed that influencing factors, adverse effects, and coping strategies were hotspots for research.

**Conclusion:**

The results shed light on the burgeoning research on depression during COVID-19, particularly the relationship between depression and public health. In addition, future research on depression during COVID-19 should focus more on special groups and those at potential risk of depression in the general population, use more quantitative and qualitative studies combined with more attention to scale updates, and conduct longitudinal follow-ups of the outcomes of interventions. In conclusion, this study contributes to a more comprehensive view of the development of depression during COVID-19 and suggests a theoretical basis for future research on public health.

## Introduction

The COVID-19 pandemic had a tremendous impact on humans, crippling daily life activities and posing a serious threat to human health, particularly regarding public mental health. The outbreak of the COVID-19 pandemic caused widespread mass panic. Meanwhile, the lockdown brought about by the COVID-19 outbreak has further triggered psychological stress among the public, including symptoms of depression, anxiety, and posttraumatic stress. For example, Wu et al. found that depression and anxiety rates were significantly higher among college students during the pandemic than before, due to factors such as being isolated at home, online learning stress, and conflict between parents and children (1). Socioeconomic instability, increased burden of living, social isolation, and unemployment were suicidal behavior triggers during the pandemic (2–4). Furthermore, fear of infection, unpredictability, and uncertainty of the COVID-19 pandemic were major stressors that triggered various mental health problems (5, 6). Thus, studying the public's mental state under the impact of a pandemic has become necessary.

The study of depression was an important area of research, even before the pandemic, focusing on depression measurement tools, influencing factors, and treatments. First, in its assessment, many researchers have developed appropriate scales, such as the Beck Depression Inventory and Montgomery Depression Test Scale. Second, regarding factors influencing the development of depression, psychosocial stress (7), patient status (8, 9), physical health status (10, 11), obesity (12), cross-cultural factors (13), and others play a role. Third, many scholars are still exploring treatments for depression, which can be divided into psychotherapy, pharmacotherapy, and other treatments (14, 15).

The relationship between depression and COVID-19 has received considerable attention in the face of sudden outbreaks. There are many theoretical and practical studies on depression during COVID-19, focusing on four aspects: the causes of depression during COVID-19, influencing factors, effects on people, and methods of alleviation. First, regarding its causes, several studies have concluded that the public's social activities were restricted due to pandemic prevention and control measures and that the masses were faced with a lack of exercise and unstable economic resources, in addition to the fear of infection (16, 17). For example, an online survey of 1,258 Italian residents revealed that social isolation during the pandemic had a significant psychosocial impact on people, especially those in vulnerable groups within the population (18). Second, various factors influenced depression during the COVID-19 pandemic. These factors can be divided into several categories: sex, age, occupation, and environmental factors, such as daily exercise. In a study involving 2,992 adults in China, depression rates were higher among men than women and higher among young adults than older adults (19). In addition to sex and age, several other factors contribute to depression. Some researchers have linked the emergence of psychological problems, such as depression, to professions (20–22). Third, depression during COVID-19 also affects public behaviors, such as insomnia (23, 24), alcohol abuse (25, 26) and irregular eating behaviors (27). Fourth, many scholars are currently seeking better ways to cope with the current trend of a high prevalence of psychological disorders, allowing for alleviation. Healthcare workers, for example, face heavy work pressure during the pandemic and usually have higher psychological stress and a higher prevalence of depression than the general population. In response, the mental burden on healthcare workers can be reduced by providing high-quality and transparent communication and accurate information updates to all staff, complete and high-quality personal protective equipment, and supplies to prevent infection (28).

This study applies an innovative approach to conducting literature reviews through systematic reviews with the support of COOC (29) and VOSviewer software, commonly used for bibliometric analysis. COOC is the most powerful bibliometrics and knowledge mapping software available, which eliminates duplication, clears multiple databases, and constructs multidimensional relationship matrices simply and efficiently (29–31). Similarly, VOSviewer is a scientific tool for creating web-based maps, visualizing and navigating them, and presenting large amounts of data in the form of knowledge maps (32). As a powerful tool for quantitatively assessing various parameters related to scientific literature published in a specific field, bibliometric analysis provides insights into popular research topics, trends, key researchers, and scientific institutions (33, 34). Several studies using bibliometric methods have been conducted in areas related to the COVID-19 pandemic. For example, Fan et al. (35) compared English and Chinese COVID-19 literature using bibliometric methods to summarize their differences and characteristics. Another study used a bibliometric approach to analyze literature related to the pediatric field during COVID-19 (36). During the last 3 years of the COVID-19 pandemic, while research in the depression-related field has evolved, the use of bibliometric methods to study this field remains incomplete (37–39).

This study provides a broad understanding of depression during COVID-19 and highlights key research topics to provide ideas for future research. It focuses on a systematic review of depression during COVID-19 using the bibliometric software COOC and VOSviewer, while considering and addressing the following questions: What are the research trends and evolutionary paths of depression during COVID-19? Which countries, authors, and institutions contributed the most to this research area? What are the research hotspots? What are the implications and limitations of this research? Considering the above, this study not only provides a deeper analysis of the literature in the field of depression during COVID-19 but also broadens the ideas for future research and provides a basis and reference for innovation in this field of research.

## Data sources and research methods

### Data sources

In this study, WOS was used as the literature information acquisition platform, and SSCI and SCIE in the core collection of WOS were used as data sources. A general search was selected, and the search conditions are shown in Table 1, with Articles, Early Access, and Review Articles selected as the literature types, and the search time range started in 2020 and ended on August 31, 2022, yielding 12,331 records. In addition, to ensure the integrity of literature retrieval, we extracted the corresponding terms from Medical Subject Headings (http://www.ncbi.nlm.nih.gov/mesh/). After merging the retrieved data with synonyms and removing duplicate and missing keyword documents, 9,464 valid records were obtained.

**Table 1:** Summary of data source and selection.

**Search settings**	**Contents**
Databases	Science citation index expanded, social sciences citation index
Search term	TS = “depression” and “COVID-19” TS = “depression” and “SARS-CoV-2” TS = “depression” and “Novel Coronavirus 2019” TS = “depression” and “coronavirus-2” TS= “depression” and “Coronavirus disease 2019” TS = “depression” and “2019-nCOV”
Language	English
Literature type	Articles, early access, review articles
Date of search	August 31, 2022
Number of records	9,464

### Research methods

Through the quantitative data analysis, bibliometrics summarizes and presents the developmental history and research hotspots of a given field. Correspondingly, based on bibliometrics, the analysis of scientific knowledge maps transforms complex knowledge from data mining and information processing into a visual knowledge map that assists scholars in scientifically obtaining the laws of dynamic development in relevant fields. Available software includes COOC, VOSviewer, CiteSpace, Bibexcel, and Bicomb. In this study, COOC and VOSviewer were mainly used, which were jointly developed by Academic Drip and the bibliometric team and are the most complete and relatively simple to operate in the bibliometric field. In addition, COOC can quickly construct relationship matrices and instantly derive matrix results such as word-part and dissimilarity matrices. More importantly, it can also pre-process data, such as batch merging synonyms and removing unnecessary words.

COOC does not currently allow for citation analysis; therefore, this study combined it with VOSviewer, developed in collaboration with Nees Jan van Eck and Ludo Waltman at Leiden University in the Netherlands, as a bibliometric analysis, and visualization tool based on a Java environment to further analyze the field of research on depression during COVID-19 (40). This is a powerful tool for “co-occurrence” network clustering and density analysis. In addition, while VOSviewer lacks data pre-processing and fast matrix generation functions compared to the COOC software, its powerful graphical presentation capabilities allow for better visualization of bibliographic relationships and provide an excellent operating environment for this study (40, 41).

**Figure 1:**
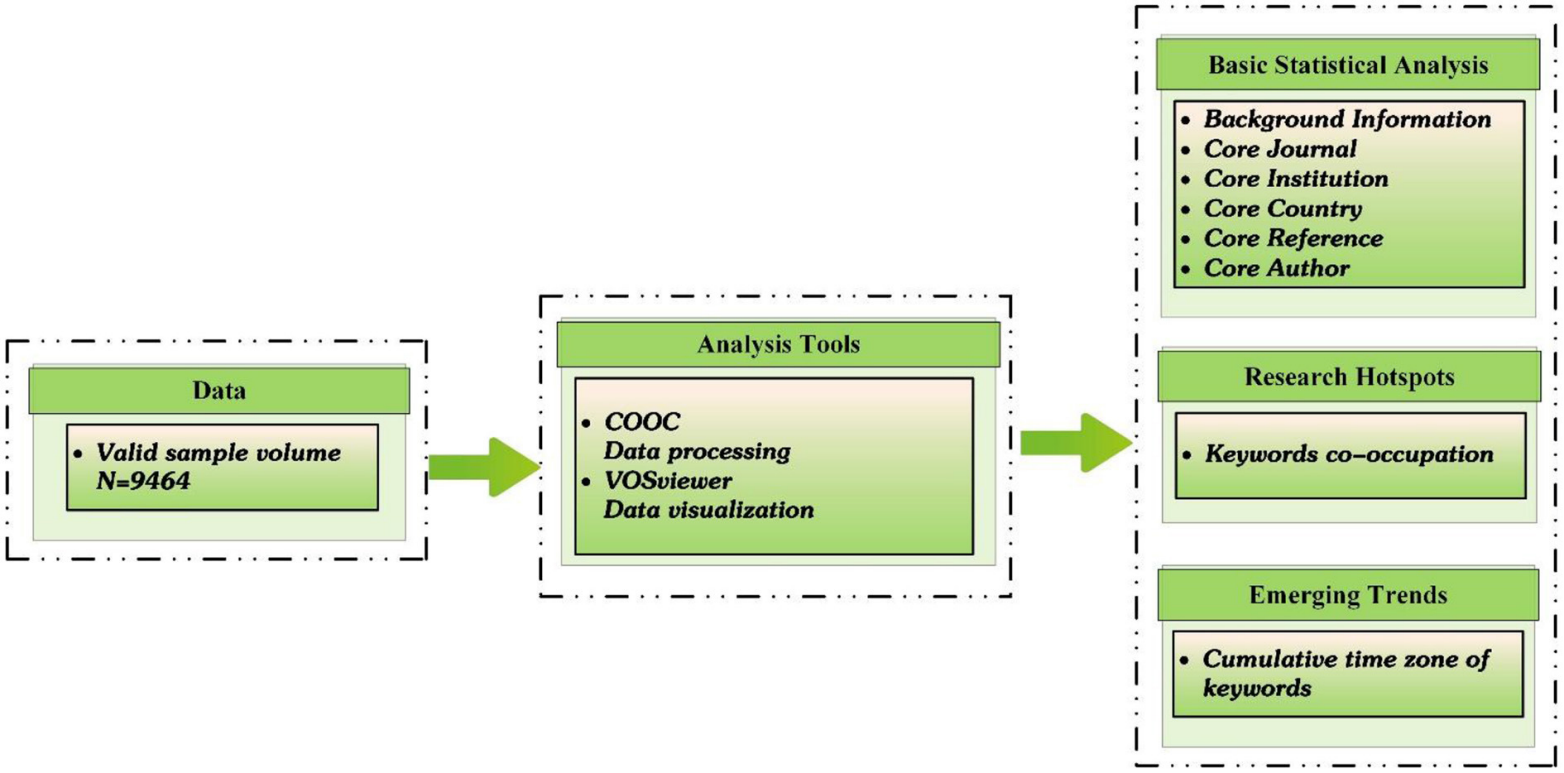
Flow chart of the research process.

This study was based on the retrieved literature and utilized tools including COOC and VOSviewer for statistical analysis, information processing, and visual knowledge mapping to comprehensively grasp the research hotspots and dynamic change patterns of “depression during COVID-19.” COOC was used to create a cumulative time-zone map, while VOSviewer was used to create a co-occurrence map, as shown in Figure 1.

## Results

### Background information

**Figure 2:**
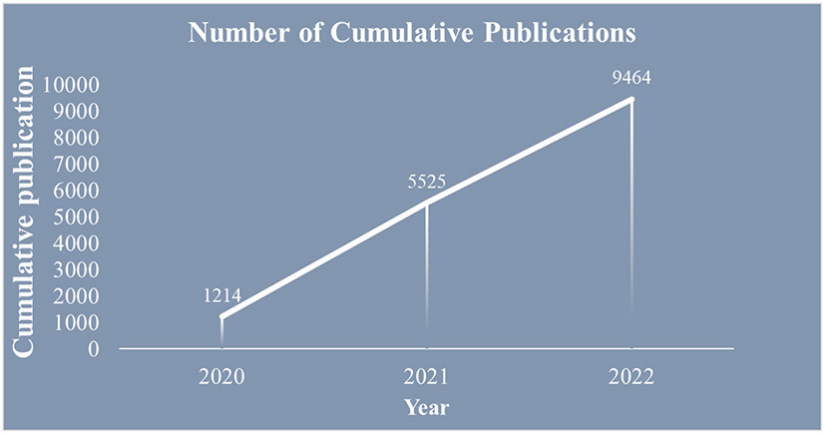
Number of cumulative publications on depression during COVID-19.

The statistics of the temporal distribution of the literature partially reflect the level of research and development in the field, as shown in Figure 2. Overall, the relevant research literature in this field started late; however, the number of publications is increasing. The COVID-19 pandemic started to spread at the end of 2019, and the number of related publications was 1,214 at the end of 2020. The cumulative number of publications reached 5,525 by 2021, with a rapid upward trend in annual publications. As of August 31, 2022, 9,464 articles had been published.

**Figure 3:**
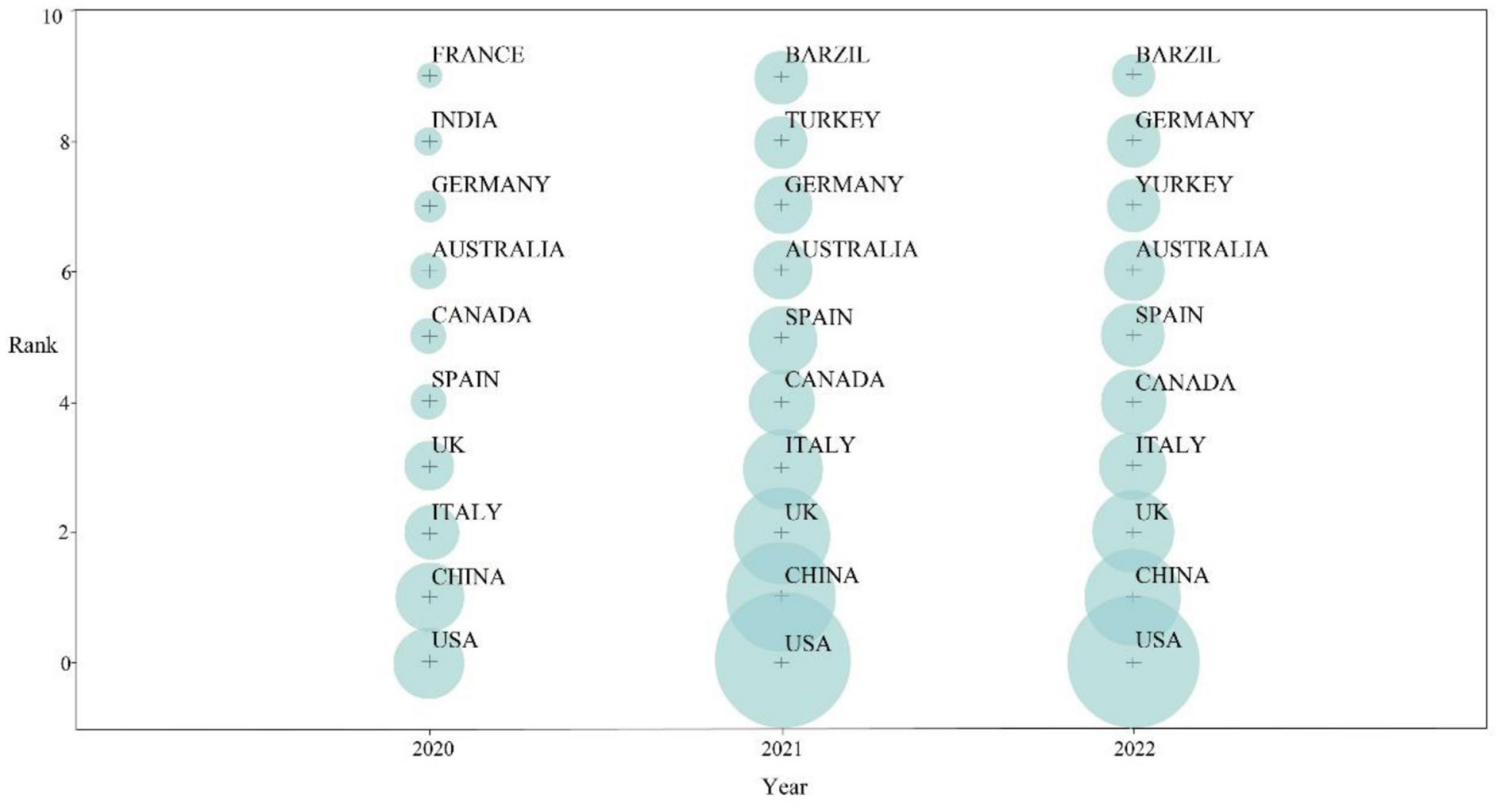
Top 10 countries with the highest number of publications from 2020 to 2022.

Importing the processed literature data in the COOC software yielded the top 10 countries with the highest number of publications from 2020 to 2022, as shown in Figure 3. Since 2020, there has been a gradual increase in research on depression during the COVID-19 pandemic. Articles published in 2020 were mainly from countries such as the United States, China, and Italy. As the COVID-19 epidemic continued to spread and worsen, the year 2021 witnessed a steady spurt of relevant literature, with 4,311 publications. Articles published in 2021 were mainly from the United States, China, the United Kingdom, Italy, Canada, and Spain. As of August 31, there were 3,939 articles published for 2022, mainly from the United States, China, and the United Kingdom.

### Core journal analysis

According to statistics, 1,727 journals are involved in depression during COVID-19 research publications at WOS, and the top 10 journals in terms of the number of articles published are shown in Table 2, accounting for 29.17% of the total literature volume. Journals with citations ≥2,924 are considered among the top 10 journals in terms of citations, while in Table 2, the International Journal of Environmental Research and Public Health, Frontiers in Psychiatry, Frontiers in Psychology, PLOS One, Journal of Affective Disorders, and Psychiatry Research are among the top 10 cited journals. These six journals can shed light specifically on research hotspots and evolutionary trends in the field of research on depression during COVID-19, which can provide direction and ideas for future research.

**Table 2:** Top 10 journals ranked by number of publications.

**Rank**	**Journal**	**Publication**	**Citation**	**Subjects covered**
1	International Journal of Environmental Research and Public Health	769	15,170	Environmental Sciences; Public, Environmental & Occupational Health
2	Frontiers in Psychiatry	492	5,943	Psychiatry
3	Frontiers in Psychology	420	6,137	Psychology, Multidisciplinary
4	PLOS One	222	6,749	Multidisciplinary Science
5	Journal of Affective Disorders	206	7,515	Clinical Neurology; Psychiatry
6	Frontiers in Public Health	179	1,200	Public, Environmental & Occupational Health
7	BMJ Open	140	1,198	Medicine, General & Internal
8	Psychiatry Research	116	7,790	Psychiatry
9	Current Psychology	110	552	Psychology, Multidisciplinary
10	Healthcare	106	580	Health Care Sciences & Services; Health Policy & Services

Statistics show that journals in the field of depression research during the COVID-19 pandemic focus on psychology, clinical practice, psychiatry, and others.

### Core institution analysis

**Figure 4:**
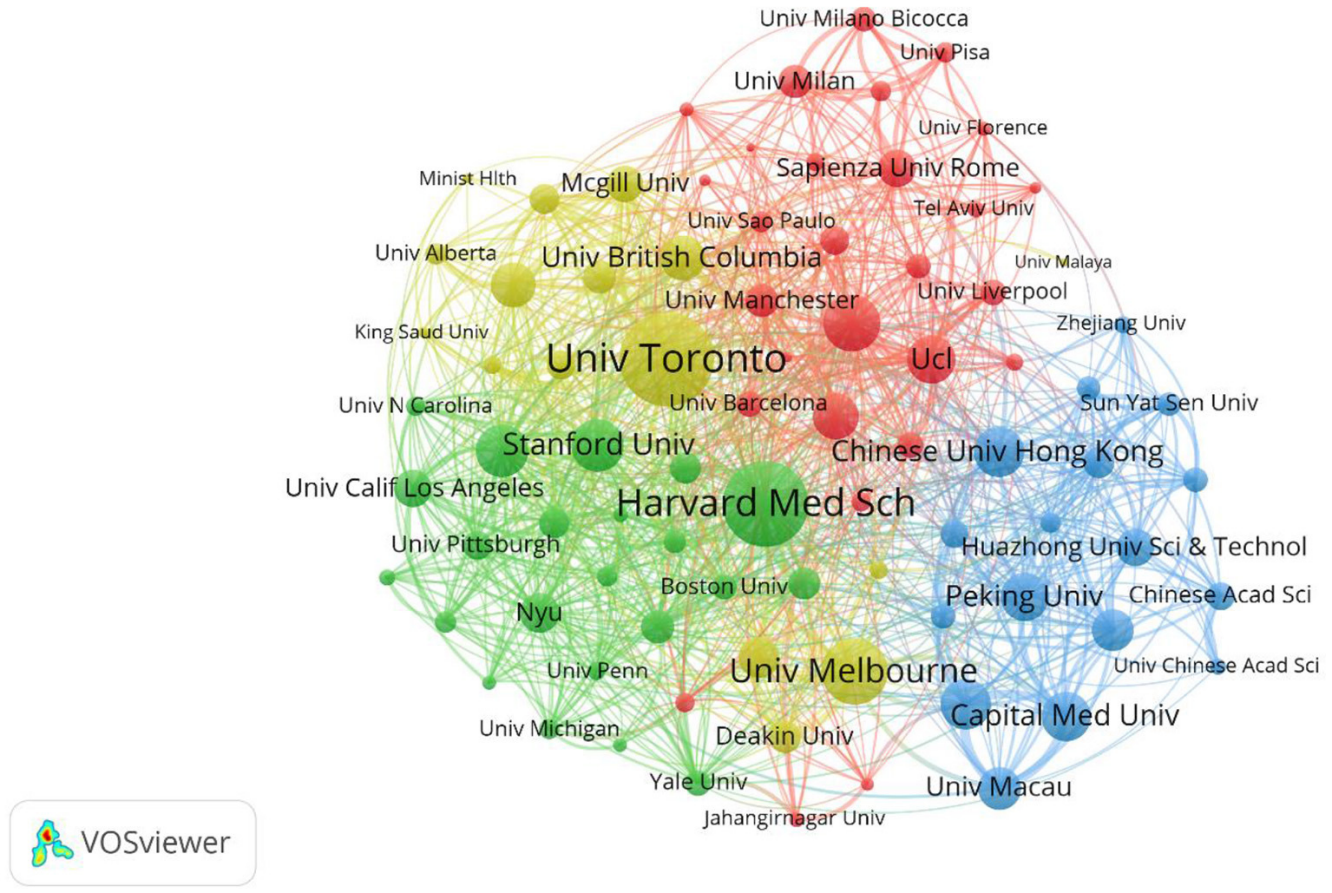
Map of institution network. The nodes represent institutions. The lines represent cooperation relationships.

To further understand the cooperation relationship between institutions, the data were imported into VOSviewer by setting the frequency to be ≥40, and the remaining parameters to default, resulting in 83 institutional cooperation networks, as shown in Figure 4. As can be seen from the inter-institutional cooperation network diagram, cooperation between institutions is relatively close. Kings College London, University of Toronto, and Harvard Med School are at the center of the network.

Table 3 shows the top 10 institutions by number of publications, and the top 10 institutions had ≥3,880 citations. Statistics show that Kings College London, University of Toronto, Wuhan University, and University College London (UCL) are among the top 10 institutions regarding the number of published articles and citations. This shows that these four institutions are in a highly significant position in the field of depression during COVID-19 research and can lead future trends and hotspots in the field. From the institutional cooperation chart and the top 10 institutions ranking table, clearly the main force in research on depression during COVID-19 is major universities. Most of these universities are from the United States, China, and the United Kingdom, where universities are more active in the research field and the connections between universities from different countries are stronger.

**Table 3:** Top 10 institutions ranked by number of publications.

**Rank**	**Institution**	**Publication**	**Citation**
1	Univ Toronto	177	7,504
2	Harvard Med Sch	162	2,771
3	Kings Coll London	149	8,727
4	UCL	139	5,050
5	Columbia Univ	121	2,494
6	Huazhong Univ Sci & Technol	118	2,973
7	Univ Melbourne	118	1,337
8	Sapienza Univ Rome	100	3,153
9	Wuhan Univ	95	6,502
10	Univ Oxford	93	2,624

### Core country analysis

The number of publications in a country reflects the level of research and impact of the country in the relevant field. Table 4 lists the top 10 countries in terms of the number of publications on depression during COVID-19. As can be seen from Table 4, the United States had the highest number of publications (2,557 times), followed by China (1,535 times), the United Kingdom (1,289 times), Italy (806 times), Canada (636 times), Spain (588 times), Australia (523 times), Germany (480 times), Turkey (412 times), and Brazil (325 times). The United States, China, and the United Kingdom have accounted for more than 50% of the publications in this field and have made major contributions to research in this area.

**Table 4:** Top 10 countries ranked by number of publications.

**Rank**	**Country**	**Publication**	**Citation**
1	United States	2,557	43,143
2	China	1,535	36,123
3	United Kingdom	1,289	17,990
4	Italy	806	13,006
5	Canada	636	10,383
6	Spain	588	10,602
7	Australia	523	8,849
8	Germany	480	8,103
9	Turkey	412	5,892
10	Brazil	325	6,384

**Figure 5:**
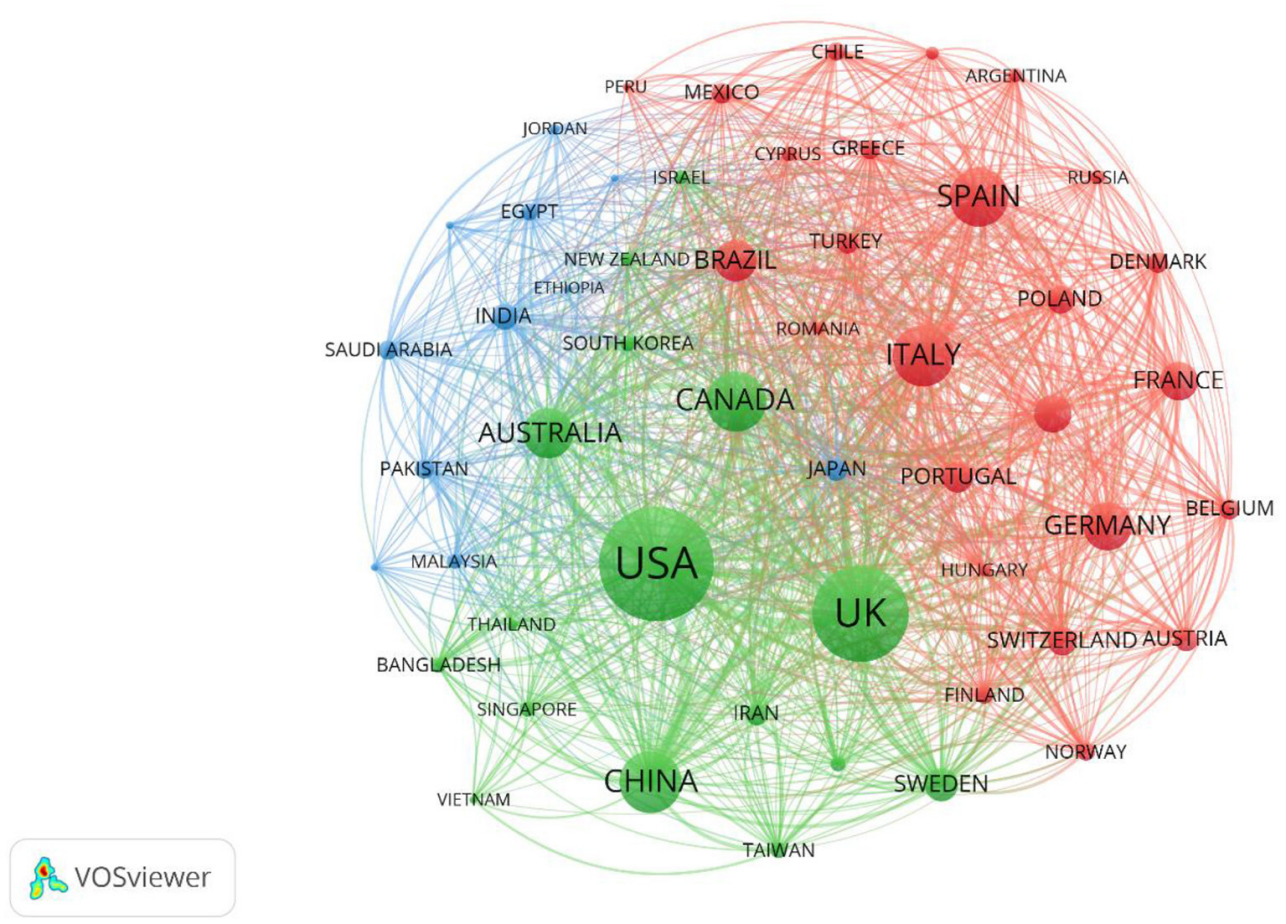
Map of countries network. The nodes represent countries. The lines represent cooperation relationships.

The retrieved literature from 144 countries was imported into VOSviewer with the frequency set to 40, and the remaining parameters defaulted to obtain the cooperation network graph from 52 countries, as shown in Figure 5. The major research forces in this field are concentrated in the United States, China, the United Kingdom, Canada, Australia, Italy, and Spain. There are cooperative relationships among various countries, particularly the United States, with China and the United Kingdom having strong ties. Analyzing cooperative exchange relations between countries is conducive to further in-depth research on depression during the COVID-19 pandemic, which is an inevitable trend in research and development in this field.

### Core reference analysis

Citation analysis can reflect the structure of research and important literature in the field. To further understand the citation structure of the depression research field during COVID-19, this study analyzed the cited literature with critical reading and obtained three clusters. The required data were imported into VOSviewer, and the frequency was set to 300 to obtain the top 30 cited studies, as shown in Table 5. The most frequently cited references were Spitzer R. L. (published in 2006; cited 1391 times), Brooks S. K. (published in 2020; cited 1351 times), Kroenke K. (published in 2001; cited 1156 times), Wang C. Y. (published in 2020; cited 1053 times), Lai J.B. (published in 2020; cited 1040 times), and Holmes E.A. (published in 2020; cited 834 times).

**Table 5:** Top 30 references with the strongest citation bursts.

**Clusters**	**References**	**Strength**	**Citations**
Mental health status, influencing factors, and coping strategies of the public during COVID-19	Ahorsu D.K., 2022, int j ment health ad, v20, p1537, doi	1,137	410
	Brooks S.K., 2020, lancet, v395, p912, doi	3,493	1,531
	Ettman C.K., 2020, jama netw open, v3, doi	875	365
	Holmes E.A., 2020, lancet psychiat, v7, p547, doi	2,453	834
	Lovibond P.F., 1995, behav res ther, v33, p335, doi	1,027	481
	Pfefferbaum B., 2020, new engl j med, v383, p510, doi	1,637	571
	Rajkumar R.P., 2020, asian j psychiatr, v52, doi	1,697	508
	Salari N., 2020, globalization health, v16, doi	1,351	449
	Torales J., 2020, int j soc psychiatr, v66, p317, doi	1,050	342
	Vindegaard N., 2020, brain behav immun, v89, p531, doi	1,502	491
	Wang C.Y., 2020, int j env res pub he, v17, doi 10.3390/ijerph17051729	3,135	1,053
	Xiong J.Q., 2020, j affect disorders, v277, p55, doi	1,931	650
	Cao W.J., 2020, psychiat res, v287, doi	2,153	663
	Hawryluck L., 2004, emerg infect dis, v10, p1206, doi	1,276	409
	Huang Y.E., 2020, psychiat res, v288, doi	2,295	658
	Mazza C., 2020, int j env res pub he, v17, doi	1,575	399
	Qiu J.Y., 2020, gen psychiat, v33, doi	2,414	687
	Wang C.Y., 2020, brain behav immun, v87, p40, doi	2,109	589
	Wang C.Y., 2020, int j env res pub he, v17, doi 10.3390/ijerph17072459	979	311
Mental health status, influencing factors and coping strategies of healthcare workers during COVID-19	Chew N.W.S., 2020, brain behav immun, v88, p559, doi	1,140	306
	Lai J.B., 2020, jama netw open, v3, doi	3,005	1,040
	Luo M., 2020, psychiat res, v291, doi	1,123	305
	Pappa S., 2020, brain behav immun, v88, p901, doi	1,927	591
	Wu P., 2009, can j psychiat, v54, p302, doi	1,011	301
	Xiang Y.T., 2020, lancet psychiat, v7, p228, doi	1,602	498
	Zhang W.R., 2020, psychother psychosom, v89, p242, doi	1,173	312
	Zigmond A.S., 1983, acta psychiat scand, v67, p361, doi	818	442
Measures of mental health status	Cohen S., 1983, j health soc behav, v24, p385, doi	942	422
	Kroenke K., 2001, j gen intern med, v16, p606, doi	2,864	1,156
	Spitzer R.L., 2006, arch intern med, v166, p1092, doi	3,508	1,391

**Figure 6:**
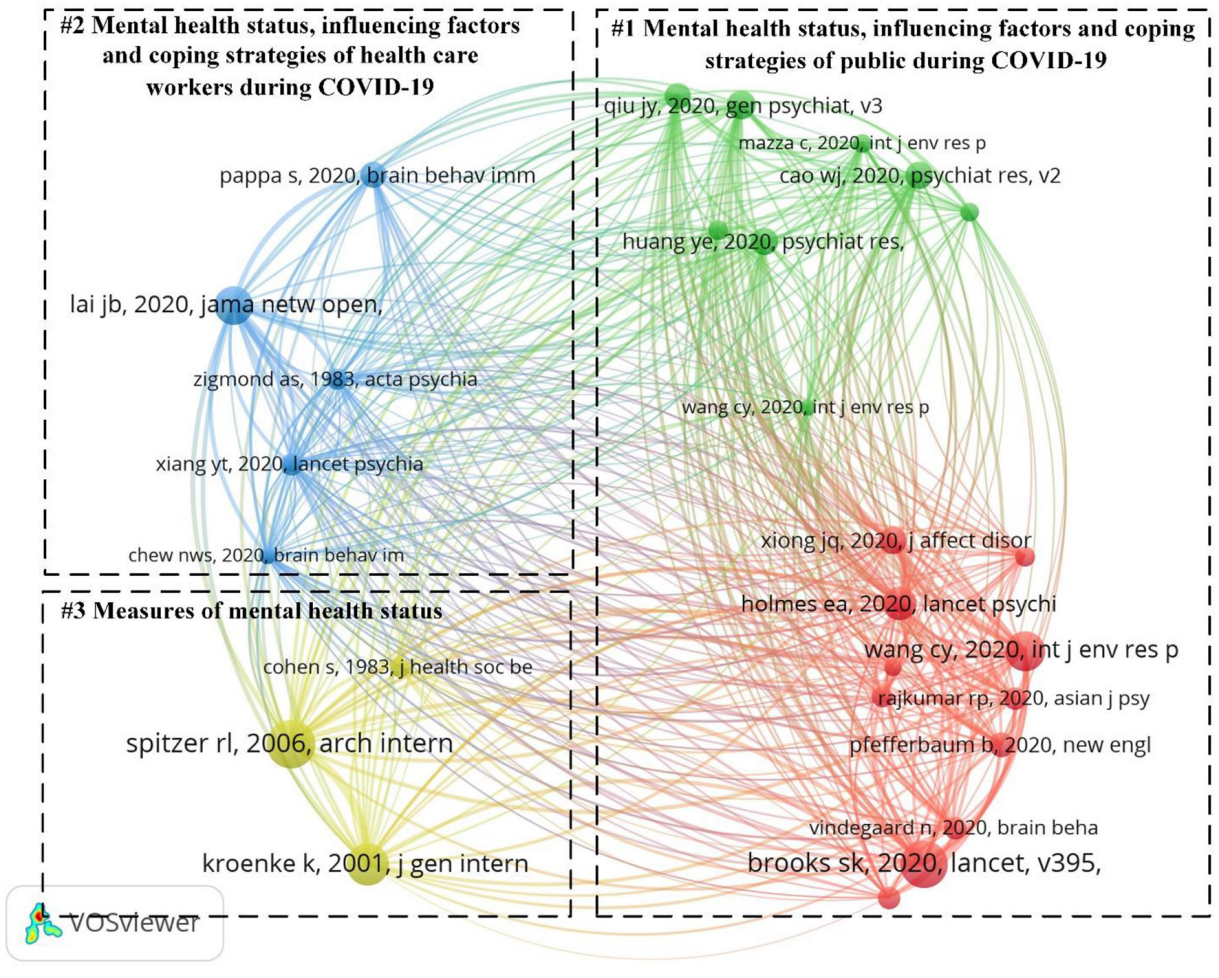
Map of references network. The nodes represent cited literature. The lines represent co-citation relationships. The green and red represent “Mental health status, influencing factors, and coping strategies of the public during COVID-19.” The blue represents “Mental health status, influencing factors and coping strategies of health care workers during COVID-19.” The yellow represents “Measures of mental health status.”

In Figure 6, the 30 references were grouped into three categories, with each color representing a category. The references with high strength values in Table 5 represent important milestones in the field of depression research during COVID-19. The first milestone was to summarize the broad psychological impact of isolation measures and to consider how to reduce this impact (42). The second milestone was to study the mental health status of healthcare workers and its associated factors during COVID-19 (43). The third milestone was to develop a validated tool to screen for generalized anxiety disorder and assess its severity (44).

### Core author analysis

**Figure 7:**
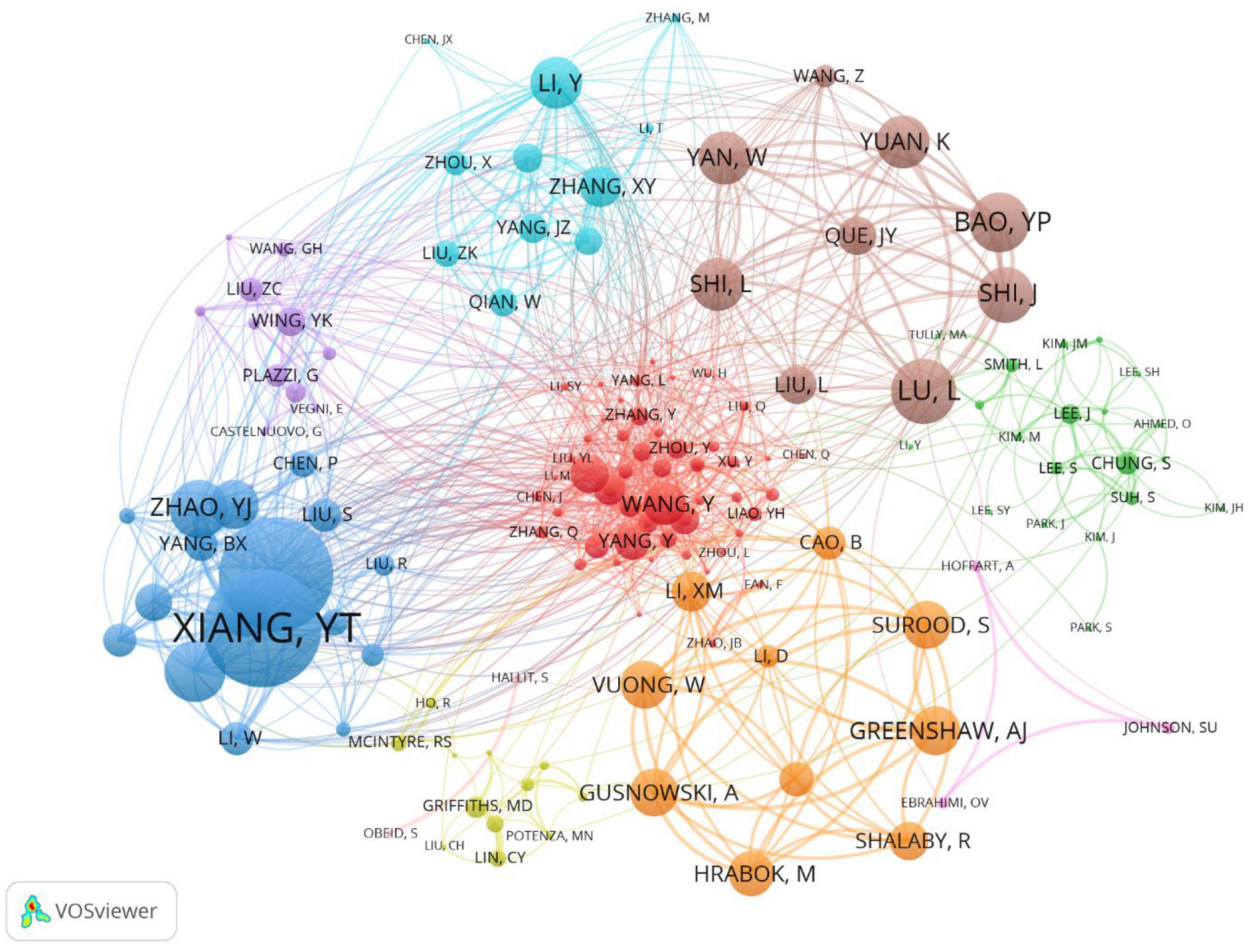
Map of co-authors network. The nodes represent authors. Node size indicates the number of articles produced. The lines represent cooperation relationships.

The number of publications by an author reflects the author's degree of influence in the relevant field. Table 6 lists the top 10 authors who published articles on depression during the COVID-19 pandemic. The statistical analysis of the authors who published articles in the field of research on depression during COVID-19 was performed using VOSviewer, with the frequency set to 10 and other parameters defaulted, to generate an author collaboration network graph, as shown in Figure 7. The nodes in the graph represent the authors, the number of articles published by the author determines the size of the nodes and fonts, and the connecting lines between the nodes represent the existence of cooperative relationships between the nodes. Overall, author collaborations in this field show a cluster-like distribution. The largest number of authors was the author collaboration network formed by Jing Li, Ying Wang, and 55 other individuals.

**Table 6:** Top 10 most productive authors ranked by number of publications.

**Rank**	**Author**	**Publication**
1	Ligang Zhang	47
2	Ying Wang	46
3	Yaya Liu	40
4	Yutao Xiang	38
5	Teris Cheung	36
6	Mark D. Griffiths	35
7	Yan Zhang	32
8	Jing Li	31
9	Xiangyang Zhang	27
10	Jianwei Wang	27

### Research hotspots

**Figure 8:**
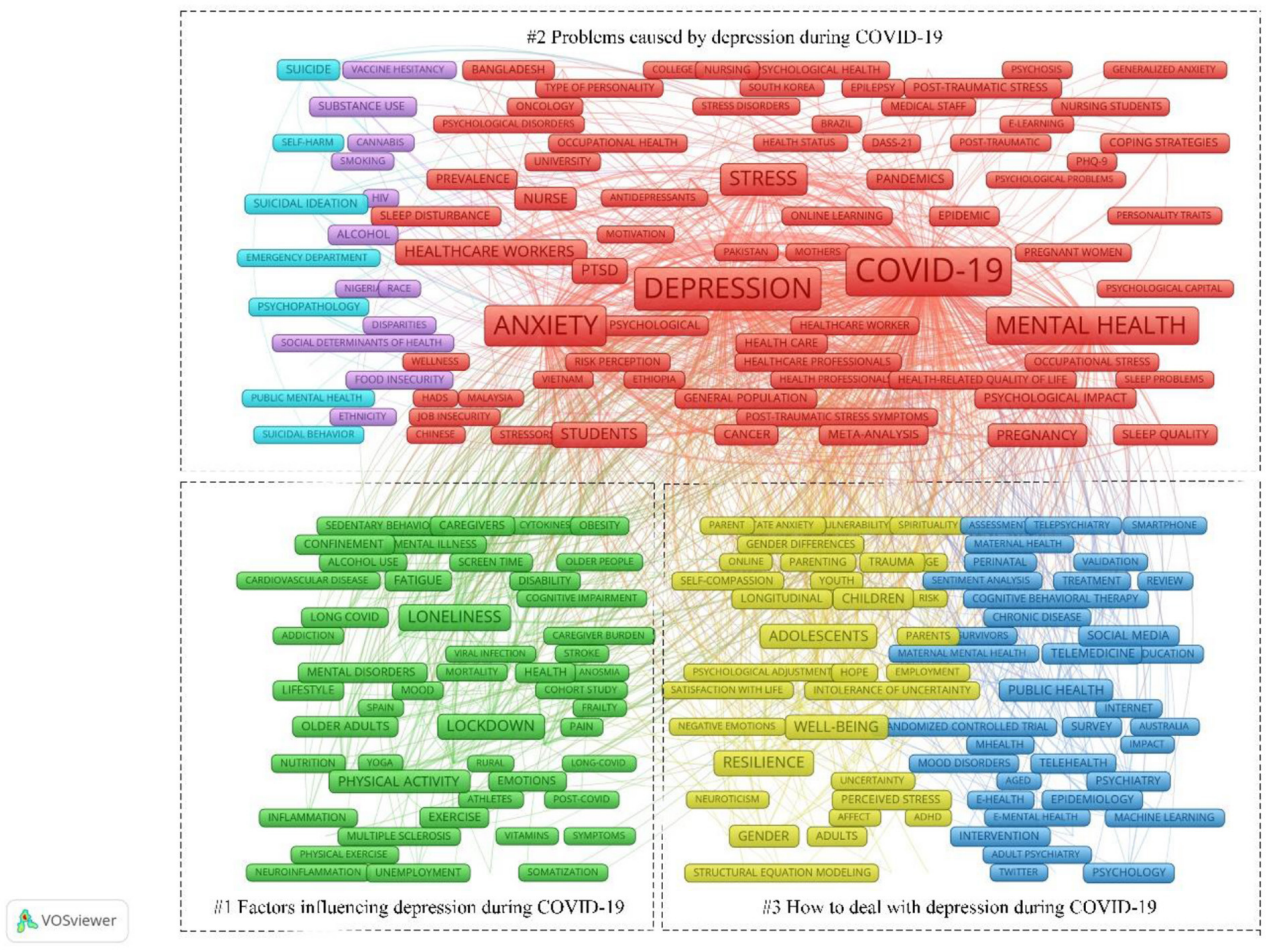
Map of keywords. The nodes represent keywords. The lines represent co-occurrence relationships. Green represents “Factors influencing depression during COVID-19.” Light blue, purple, and red represent “Problems caused by depression during COVID-19.” Dark blue and yellow represent “How to deal with depression during COVID-19.”

Keywords are the essence and distillation of the article's content, which can effectively reflect the research content, purpose, method, object, and results of the article and link them together to reveal the general lineage of the article. If a keyword appears frequently and repeatedly in a research field during a certain period, the research topic characterized by that keyword is considered the research hotspot of that research field. Table 7 lists the top 30 keywords related to the depression research field during the COVID-19 period, and the co-occurrence graph of keywords was obtained by importing the data into VOSviewer, as shown in Figure 8, the larger the corresponding font and node, the greater the weight of its keywords. The keywords “mental health,” “anxiety,” “lockdown,” and “adolescents” are important hotspots in depression-related fields during COVID-19. Through the analysis, the following three main categories of research hotspots on depression during COVID-19 were obtained.

**Table 7:** Top 30 highest frequency keywords related to depression during COVID-19.

**Keywords**	**Frequency**	**Keywords**	**Frequency**
COVID-19	8,651	Physical activity	187
Depression	3,708	Depressive symptoms	187
Anxiety	2,864	Burnout	182
Mental Health	2,204	Insomnia	177
Stress	933	Quarantine	175
Loneliness	499	Public health	171
Resilience	356	Pregnancy	168
Students	345	Sleep	160
Lockdown	338	Nurse	160
PTSD	310	Older adults	132
Healthcare workers	297	Risk factors	129
Wellbeing	282	Gender	119
Adolescents	274	Children	113
Quality of life	240	Psychological impact	102
Social support	205	Suicide	98

#### Factors influencing depression during COVID-19

High-frequency keywords included in the study's hotspots were lockdown (338 times), healthcare workers (297 times), physical Activity (187 times), burnout (182 times), older adults (132 times), gender (119 times), and unemployment (25 times). During the COVID-19 pandemic, the prevalence of mental illness was significantly higher than before the pandemic. Studies on the prevalence of depression during the pandemic have focused on factors that influence mental illness. Various analytical methods have been used to investigate this issue, and the following factors have been identified:

1. Quarantine policies enacted by governments during the COVID-19 pandemic reduced exercise time and social activities for the population in each country, and the lack of both activities was a significant factor in the elevated prevalence of mental illness in the population (45).
2. During the COVID-19 pandemic, most productive social activities were halted, and the country's population was left with unemployment and job insecurity (46, 47).
3. The sex, age, occupation, and social media use of residents influenced the development of mental illness (47–49).

#### Problems caused by depression during COVID-19

High-frequency keywords included in this research hotspot were PTSD (310 times), quality of life (240 times), insomnia (177 times), sleep (160 times), suicide (98 times), and alcohol (47 times). During the pandemic, mental illness has also caused problems such as insomnia, alcoholism, suicide, and smartphone addiction (50–52). These seriously affect the quality of life of people, especially adolescents (53); teenagers, college students, older adults (54); health care workers (55–57); and pregnant women (58, 59).

#### How to deal with depression during COVID-19

The high-frequency keywords included in this research hotspot were resilience (356 times), well-being (282 times), and social support (205 times). The increase in the prevalence of mental health problems, particularly depression, during the COVID-19 pandemic, cannot be ignored; therefore, how should this phenomenon be faced and what measures should be taken to prevent or alleviate it? Some scholars suggest that the phenomenon of “social isolation” caused by home isolation can be alleviated through digital media (60). Additionally, frontline healthcare workers should improve their subjective wellbeing and pay attention to their mental health status (61). For those who are already depressed, interventions to promote resilience should be provided whenever possible (62–64). In addition, authorities should provide adequate supplies to the population during the quarantine period and promote the benefits of public isolation to society.

### Emerging trends

**Figure 9:**
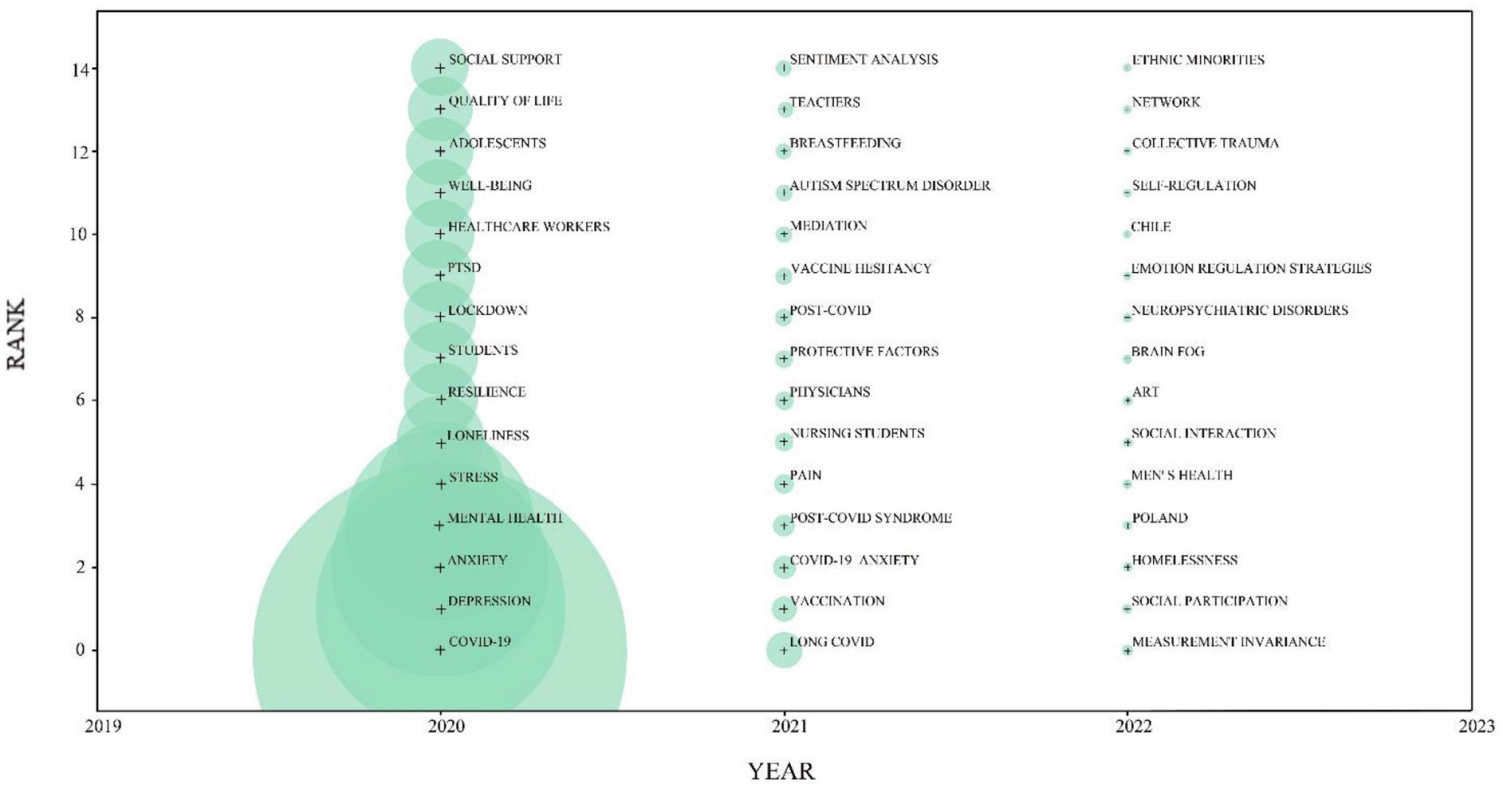
Emerging trends of research on depression during COVID-19. The nodes represent keywords. The node size indicates the number of keyword occurrences. The year corresponding to the node is the year in which the keyword first appeared.

The cumulative time zones of the keywords were mapped using COOC, and Figure 9 shows the top 15 high-frequency keywords for 2020–2022. The graph provides an overall picture of changes in the study path (65–68). The size of the circles next to the keywords represents the number of keyword occurrences. The 2020 study concluded that the outbreak and prolonged isolation caused by the COVID-19 pandemic would affect public mental health, particularly the wellbeing of adolescents and healthcare workers, and emphasized that negative emotions should be alleviated through social support and improving psychological resilience. The 2021 study concluded that the duration of the COVID-19 pandemic was long. The 2022 study focused on the harm caused by depression during COVID-19, such as economic collapse, loss of fixed housing, brain fog, neuropsychiatric disorders, and collective trauma, as well as on coping strategies, including social interaction, social engagement, and good mood regulation strategies.

## Discussion

### Discussion of the results

In this study, WOS was selected as a search platform for bibliometric analysis of publications, countries, institutions, and keyword counts in the field of depression during COVID-19 and for the presentation of scientific knowledge maps. Statistics show that the literature in this field peaked in 2021. In total, 144 countries, 48,103 authors, and 10,853 research institutions worldwide participated in this study. Of these, the United States, China, and the United Kingdom had the highest total number of publications and strong collaborative relationships, and the main research institutions in each country are universities. In addition, this study specifically focused on exploring the evolution of knowledge structures and research themes. Regarding the development of this study and its hotspot tracking, the important findings are as follows:

First, a general upward trend in research on depression during COVID-19 is evident in terms of research progress. Although this area has been studied for <3 years, it has received widespread attention worldwide due to its specificity. The number of publications in the field of depression during COVID-19 peaked in 2021, and related research in this field entered a period of rapid development. This may be because several studies have found significant changes in the frequency of public psychological problems arising at two time points, before the pandemic and during the embargo. In particular, the prevalence of depression was greatly increased during the embargo (1, 69–74). Moreover, according to the World Health Organization, the pandemic has led to a significant increase in the global prevalence of depression and anxiety disorders by 28 and 26%, respectively (75). COVID-19 has significantly impacted global healthcare, and new research hotspots are gradually shifting from COVID-19 and related clinical studies to studies on its psychological and social impact on humanity (36). Therefore, the shift in research hotspots and the societal impact of COVID-19 have been influenced by COVID-19's significant impact on global healthcare. As a result, an increasing number of scholars have started to conduct research on depression during the COVID-19 pandemic, influenced by the shift in research hotspots and social concerns.

Second, the hotspots of depression research during COVID-19 were significantly concentrated in regions such as the Americas, Western Europe, and South Asia. This may be related to the severity of the COVID-19 pandemic in each region. For example, India, which is in South Asia and had the second-highest total number of people diagnosed with COVID-19 worldwide, had a high prevalence of psychological problems among the population during the COVID-19 pandemic (76). In addition, a study on adults from several countries showed that participants living in Brazil had the most severe symptoms of anxiety and depression (77). This may be related to the severe COVID-19 pandemic experienced in Brazil. Furthermore, several Western European countries have paid considerable attention to the impact of the COVID-19 pandemic on public mental health and have conducted corresponding studies. For example, Bäuerle et al. found that the public mental health burden in Germany significantly increased during the COVID-19 pandemic (78). Notably, panic after infection, social isolation during treatment, and implicit discrimination after recovery were important causes of depression during COVID-19 in confirmed individuals (79, 80).

Finally, the hotspots of depression research during the pandemic could be summarized into three areas: factors influencing depression during COVID-19, consequences of depression during COVID-19, and coping strategies for depression during COVID-19.

#### Factors influencing depression during COVID-19

To better explain the relationship between depression and COVID-19, the factors influencing the formation of depression during COVID-19 were examined. Regarding social factors, the pressure from rising house prices (81), negative news spread on social media (82), the anxiety caused by social isolation (83, 84), and others are contributing factors to the poor mental health of residents during the pandemic. First, the ecosystem theory proposed by Bronfenbrenner mainly studies the interrelationship between human behavior and the social environment, which treats the social environment in which humans grow up as a kind of social ecosystem, emphasizes the importance of the ecosystem for analyzing and understanding human behavior, and focuses on the interaction between humans and environmental systems and their influence on individual development. Social factors that lead to depression during COVID-19, such as rising housing prices, negative news, and social isolation, change the social environment on which human growth depends. Changes in the social environment inevitably affect individual development and, consequently, have an impact on public mental health.

Regarding characteristic factors, gender, age, and occupation of the residents are conditions for the occurrence of depression during COVID-19 (85). For example, several meta-analytic studies on the prevalence of depression during COVID-19 among frontline healthcare workers have shown that they exhibit higher levels of depression than the general population (86, 87). During the pandemic, healthcare workers not only face a heavy workload but also the risk of contracting COVID-19, and their negative emotions need to be attended to. Additionally, a meta-analytic study by Li et al. found that the prevalence of depression and anxiety disorders among college students has greatly increased (88). According to Erikson's theory of psychological development, college students are still in the sameness-to-role confusion stage, and their psychological development is still in a transitional stage, immature, and vulnerable to disturbance by negative external events. Moreover, during the pandemic, college students are forced to be isolated at home, which interrupts their social activities. They have to also manage their online studies, and all of these contribute to their depressive symptoms.

#### Problems caused by depression during COVID-19

Brailovskaia et al. found a significant increase in suicidal ideation and suicide rates among the public during the COVID-19 pandemic (89). The American clinical psychologist Beck (90) proposed a cognitive model of depression that argues that the underlying cognitive schema and cognitive theoretical assumptions of depressed individuals are at the root of patients' negative attitudes. Some individuals with depression may be less depressed; however, sudden negative events in their lives give them a heavy blow, leading to further despair and helplessness. Despair and helplessness are important factors influencing suicide. Therefore, the COVID-19 pandemic, as a sudden negative life event, may be the root cause of helplessness and negative attitudes in depressed patients. Furthermore, several studies have pointed out that the probability of abnormal behaviors, such as insomnia and irregular diet, has increased significantly during the pandemic (91, 92). These studies are consistent with the Theory of Planned Behavior, which suggests that people's intentions or behaviors can be manipulated by attitudes, subjective norms, and perceived behavioral control to target behaviors (93). The negative, desperate, and helpless attitude of depressed individuals can lead to changes in their behavior, which may include loss of appetite, overeating, insomnia, and other abnormal behaviors. The rise in the probability of alcoholism and Internet addictive behavior is also a prevalent feature of COVID-19 (94, 95). Learned helplessness theory suggests that uncontrollable negative events are an important cause of depression. If such negative events are frequent and prolonged, they can lead to an uncontrollable perception that, no matter what one does, one cannot change the outcome. Because of the prolonged preventive and control measures and socioeconomic impacts during the COVID-19 pandemic, people's normal social activities are restricted, and their physical and mental health are damaged, making them prone to psychological burdens and causing them to develop learned helplessness. Alcoholism and Internet addiction are among the behaviors that make patients give up on their efforts and paralyze them.

#### How to deal with depression during COVID-19

To mitigate the increased prevalence of depression during COVID-19, treatment interventions should be improved (96, 97). Previous studies found that digital socialization, social support, and welfare measures are important in alleviating depression during the COVID-19 pandemic (98). Rose and Rudolph reconstructed the interpersonal context theory based on the interpersonal theory (99), arguing that negative early family experiences can cause individuals to develop negative interpersonal relationship evaluation tendencies and social behavior disorders, which adversely affect subsequent interpersonal skills and thus deepen public depression. Therefore, residents can reduce the incidence of depression during COVID-19 by constructing good interpersonal relationships. House (100) considers social support as an interpersonal transaction involving emotional care (liking, love, empathy), instrumental assistance (goods or services), information (environment), and assessment (information related to self-assessment). In sociological theory, Virginia (101) states that social support is a reciprocal relationship between individuals and networks that provide psychological, social, and substantive help through social networks. Therefore, to some extent, social support can alleviate the development of depression during COVID-19.

In addition, residents can prevent depression by exercising regularly and actively adjusting their mindset (102, 103). The embodied cognition theory emphasizes the interaction between cognitive processes and the anatomical structure of the body, body movements, and the external environment of the body (104). It is believed that cognition is not only related to the brain, but also to the body, which is the carrier of cognition and cognitive functions. The body can also directly participate in mental processes such as emotion and thinking. Lack of physical exercise, as mentioned earlier, is one of the factors contributing to depression during COVID-19. However, regular exercise can influence the mental health of the public by improving their mood.

### Implications

The significance of this study is reflected in the following two aspects: First, this study used COOC (29) and VOSviewer (40) software as tools to conduct a literature econometric analysis and scientific knowledge mapping in the field of depression research during COVID-19, aiming to systematically summarize the trends and research hotspots in this field. Second, as an emerging topic, depression during COVID-19 has not been developed for a long time; however, it has been widely noticed worldwide owing to its specificity (43, 105). In addition, this study adopted a visualization method to quickly locate the key research results in this field. A review of the literature in this area will assist future researchers in further analyzing the causes of depression and finding measures to alleviate depression during COVID-19. It also assists the government in improving the current trend of frequent public psychological problems and provides ideas and references for solving public psychological problems caused by global issues in the future (106, 107).

### Limitations and directions for future research

This study has some limitations. First, as an emerging hot topic, the earliest literature on depression during COVID-19 was published in 2020, which was <3 years ago, and scholars' research in this field is limited to the initial stage, which does not perfectly reflect the development and evolutionary trends of this field. Future research should continue to track the literature in this field over the next few years to enrich the research trends and hotspots. Second, the search platform of this study was limited by the WOS platform, the type of literature is specified, and the amount of literature obtained is incomplete. Future research should attempt to join other search platforms, such as PubMed, and compare and analyze the literature retrieved by WOS and PubMed to summarize the patterns.

Through bibliometric analysis and scientific knowledge mapping of the field of depression research during COVID-19, the following aspects may also be of interest in the future. First, most articles in the field of depression research during COVID-19 have been studied using quantitative research methods, and few have been studied using a mixture of qualitative and quantitative research methods (108, 109). Second, by reading the articles, it was established that most of the scales used to assess depression in the field articles were developed before the pandemic. Future research could thus update the depression assessment scales (110). Third, there is a lack of research on the differences in depression during COVID-19 caused by the cross-cultural context (13). Fourth, regarding factors that shape depression during COVID-19, future research should consider multi-layer linear modeling. Feinberg et al. (111) used HLM methods to study effects of public health interventions on families and individuals; in terms of causing problems, there could be a sustained focus on the specific impact of depression on special groups during COVID-19 (112); in terms of coping strategies, longitudinal studies of interventions (113) are warranted, while government (114) and society should also pay sustained attention to those with low levels of depression but are potentially at risk.

## Conclusion

This study used COOC and VOSviewer tools for a comprehensive follow-up and visual analysis of the literature in the field of depression during COVID-19. The goal of this study was to systematically review the literature in this area and draw the following conclusions. First, regarding research progress, the field of depression during COVID-19 has been studied for <3 years but has entered a rapid development period. Second, the number of regional publications in the area is related to the severity and importance of the pandemic in each region. Among these, the strongest collaboration is between the United States, China, and the United Kingdom. Finally, regarding research hotspots, the field of depression during COVID-19 is particularly concerned with “factors influencing depression during COVID-19,” “consequences of depression during COVID-19,” and “coping strategies for depression during COVID-19.” The three areas of “depression during COVID-19” are discussed. Finally, this series of studies on COVID-19 provides a reference for future exploration of public mental health in the context of a global pandemic, as well as helps to forge new pathways for addressing the legacy of human psychological problems after the end of the COVID-19 pandemic and setting a research agenda for future investigations.

## Data availability statement

The original contributions presented in the study are included in the article/supplementary material, further inquiries can be directed to the corresponding author.

## Author contributions

JZ, QF, JG, and YX designed the study. QF, JG, and YX performed the analysis and interpreted the data. JZ, XL, YY, YM, and SS reviewed the article and provided comments or suggestions. JZ had primary responsibility for final content. All authors contributed to manuscript and approved the submitted version.

## Funding

This study was supported by National Office for Education Sciences Planning, Grant Number BAA180017.

## Conflict of interest

The authors declare that the research was conducted in the absence of any commercial or financial relationships that could be construed as a potential conflict of interest.

## Publisher's note

All claims expressed in this article are solely those of the authors and do not necessarily represent those of their affiliated organizations, or those of the publisher, the editors and the reviewers. Any product that may be evaluated in this article, or claim that may be made by its manufacturer, is not guaranteed or endorsed by the publisher.
